# Peripheral blood DNA methylation and neuroanatomical responses to HDACi treatment that rescues neurological deficits in a Kabuki syndrome mouse model

**DOI:** 10.1186/s13148-023-01582-x

**Published:** 2023-10-27

**Authors:** Sarah Jessica Goodman, Teresa Romeo Luperchio, Jacob Ellegood, Eric Chater-Diehl, Jason P. Lerch, Hans Tomas Bjornsson, Rosanna Weksberg

**Affiliations:** 1 Genetics and Genome Biology, Hospital for Sick Children, Toronto, Canada; 2 McKusick-Nathans Department of Genetic Medicine, Johns Hopkins University School of Medicine, Baltimore, USA; 3 Mouse Imaging Centre (MICe), Hospital for Sick Children, Toronto, Canada; 4 Department of Medical Biophysics, University of Toronto, Toronto, Canada; 5 Wellcome Centre for Integrative Neuroimaging, The University of Oxford, Oxford, UK; 6 Nuffield Department of Clinical Neuroscience, The University of Oxford, Oxford, UK; 7 Department of Pediatrics, Johns Hopkins University School of Medicine, Baltimore, USA; 8 Faculty of Medicine, University of Iceland, Reykjavík, Iceland; 9 Landspitali University Hospital, Reykjavík, Iceland; 10 Division of Clinical and Metabolic Genetics, Hospital for Sick Children, Toronto, Canada; 11 Department of Molecular Genetics, University of Toronto, Toronto, Canada; 12 Institute of Medical Science, University of Toronto, Toronto, Canada; 13 Department of Paediatrics, University of Toronto, Toronto, ON Canada

## Abstract

**Background:**

Recent findings from studies of mouse models of Mendelian disorders of epigenetic machinery strongly support the potential for postnatal therapies to improve neurobehavioral and cognitive deficits. As several of these therapies move into human clinical trials, the search for biomarkers of treatment efficacy is a priority. A potential postnatal treatment of Kabuki syndrome type 1 (KS1), caused by pathogenic variants in *KMT2D* encoding a histone-lysine methyltransferase, has emerged using a mouse model of KS1 (*Kmt2d*^+*/βGeo*^). In this mouse model, hippocampal memory deficits are ameliorated following treatment with the histone deacetylase inhibitor (HDACi), AR-42. Here, we investigate the effect of both *Kmt2d*^+*/βGeo*^ genotype and AR-42 treatment on neuroanatomy and on DNA methylation (DNAm) in peripheral blood. While peripheral blood may not be considered a “primary tissue” with respect to understanding the pathophysiology of neurodevelopmental disorders, it has the potential to serve as an accessible biomarker of disease- and treatment-related changes in the brain.

**Methods:**

Half of the KS1 and wildtype mice were treated with 14 days of AR-42. Following treatment, fixed brain samples were imaged using MRI to calculate regional volumes. Blood was assayed for genome-wide DNAm at over 285,000 CpG sites using the Illumina Infinium Mouse Methylation array. DNAm patterns and brain volumes were analyzed in the four groups of animals: wildtype untreated, wildtype AR-42 treated, KS1 untreated and KS1 AR-42 treated.

**Results:**

We defined a DNAm signature in the blood of KS1 mice, that overlapped with the human KS1 DNAm signature. We also found a striking 10% decrease in total brain volume in untreated KS1 mice compared to untreated wildtype, which correlated with DNAm levels in a subset KS1 signature sites, suggesting that disease severity may be reflected in blood DNAm. Treatment with AR-42 ameliorated DNAm aberrations in KS1 mice at a small number of signature sites.

**Conclusions:**

As this treatment impacts both neurological deficits and blood DNAm in mice, future KS clinical trials in humans could be used to assess blood DNAm as an early biomarker of therapeutic efficacy.

**Supplementary Information:**

The online version contains supplementary material available at 10.1186/s13148-023-01582-x.

## Introduction

Kabuki syndrome type 1 (KS1) is a Mendelian disorder of epigenetic machinery (MDEM) that results from pathogenic variants in *KMT2D*, a gene encoding a histone H3 lysine 4 (H3K4) methyltransferase [1, 2]. This disorder is characterized by a common set of dysmorphic facial features (e.g., long palpebral fissures with eversion of the lateral third of the lower eyelid; arched and broad eyebrows; short columella), mild-to-moderate intellectual disability, and postnatal growth deficiency. KS1 is also associated with congenital heart defects, increased susceptibility to infections, seizures, and feeding problems [2, 3]. To manage health problems associated with KS1, individuals are followed closely from birth by health care providers to monitor for possible problems or complications and are treated after symptoms arise. Until recently, the idea of an effective postnatal treatment for neurodevelopmental disorders to pre-empt symptoms including developmental delay and intellectual disabilities in disorders such as KS1 seemed highly implausible or at least, remote.

Animal models of KS1 have not only allowed for a deeper understanding of pathophysiology but also provided foundational evidence for effective postnatal treatment of intellectual disability. Bjornsson et al. previously characterized a mouse model of KS1, *Kmt2d*^+*/βGeo*^, for which multiple treatments with drugs with epigenetic modes of action were effective in ameliorating neurological phenotypes. This mouse model recapitulates multiple features of KS1 including facial dysmorphism, learning deficits, immune dysregulation and has been characterized as exhibiting global loss of histone H3K4 trimethylation (H3K4me3) in vitro and in vivo [4–6] and aberrant chromatin accessibility [7]. AR-42, a histone deacetylase inhibitor (HDACi), improved hippocampal learning and memory deficits in *Kmt2d*^+*/βGeo*^ mice, as well as normalized H3K4me3 levels in the dentate gyrus [4]. Inhibition of KDM1A using TAK-418, a histone demethylase that removes H3K4 methylation, or increasing endogenous HDACi beta-hydroxybutyrate through a ketogenic diet led to similar phenotypic rescues in this mouse model [5, 6]. These findings have precipitated clinical trials of dietary interventions as a treatment for cognitive deficits in individuals with KS1 (Identifier NCT04722315).

In many MDEM, DNA methylation (DNAm) has proven to be an effective diagnostic tool in the research and clinical settings [8–11]. Our group published a DNAm signature of KS1 in 2017, finding this pattern of methylation to be distinct from CHARGE syndrome (a differential diagnosis) but not Kabuki type 2, caused by pathogenic variants in the X-linked gene *KDM6A* [12]. Other KS signatures have also been defined, demonstrating similar findings with specificity for the KS phenotype [13, 14]. Both KMT2D and KDM6A act to promote open chromatin by depositing activating H3K4me or removing repressive H3K27me2/3, respectively [15–17]. The human KS1 signature currently serves multiple functions including classifying variants of uncertain significance as pathogenic or benign and providing a set of loci at which transcriptional dysregulation may be contributing to the KS1 phenotype and severity [12, 13, 18]. However, there may be even greater potential for DNAm signatures as it is possible that these may work as a therapeutic biomarker capturing information of sub-phenotype presence/absence and treatment efficacy. In drug trials of treatments for neurological outcomes, biomarkers raise the success of these trials from 11 to 40% [19].

Here, we establish a KS1 mouse signature in blood DNA and assess whether this signature can serve as a biomarker of (1) phenotypic severity as measured by brain volume, and (2) treatment efficacy of AR-42. We find that the KS1 mouse signature maps to genes that exhibit DNAm aberrations in humans with KS1. Additionally, a subset of signature sites are predictive of brain volume, while a different subset exhibits a correction of DNAm levels in the AR-42 treated KS1 mice, i.e., a shift toward normal wildtype (WT) DNAm levels. Moreover, previously published H3K4me3 levels primarily showed unidirectional changes, with genome-wide loss of H3K4me3 in the *Kmt2d*^+*/βGeo*^ mice and genome-wide gain of H3K4me3 in response to AR-42 treatment [4]. Our corresponding DNAm data exhibited bidirectional changes, with no bias toward gain or loss of methylation with this genotype or following AR-42 treatment. While a simplified model of epigenetic cross-talk may predict gain of H3K4me3 to correspond to a loss of DNAm and vice versa, we saw no such relationship, highlighting the complexity of epigenetic regulation and downstream effects of disruption of transcriptional programs resultant from epigenetic perturbations. This work supports a new area of exploration for DNAm signatures, as biomarkers of treatment efficacy.

## Methods

### Animals

Methods for treatment with AR-42 follow those published in Bjornsson et al. [4]. Briefly, the KS1 mouse model, *Kmt2d*^+*/βGeo*^ was acquired from Bay Genomics (University of California) but have since been fully backcrossed onto a pure C57BL/6J background. Mice were housed at the Johns Hopkins Mouse Facility. Animals in this study were born across 9 litters resulted from timed matings within a 3–4 day window, and assorted in genotype and sex matched pairs to cage, and treatment group. Only males were used in this experiment in an effort to provide sex-disaggregated data while also being limited by experimental time and cost constraints. Mice were dosed by oral gavage daily beginning at postnatal week 4 for 2 weeks, with vehicle alone (0.5% methylcellulose, 0.1% Tween-80, water) or treated with vehicle + 10mg/kg AR-42 (Selleck Chemicals). Cages were randomly selected for sacrifice one or two days after the end of treatment. Mice were weighed and blood was collected from the submandibular vein into K2EDTA-coated microtainer tubes (lavender top) for epigenetic analysis. Mice were anesthetized with a lethal dose of ketamine/xylazine and prepared by transcardiac perfusion (first flush: PBS + 1USP/mL heparin + 2mM ProHance, second flush: 4%PFA + 2mM ProHance), and skulls were stored in 4% PFA + 2mM ProHance overnight. Buffer was replaced with 1xPBS + 0.02% sodium azide + 2mM ProHance and stored 4C until imaging. As all samples did not meet quality control, experiments differ in their sample sizes despite being run on “matched” tissues (Table 1). All experiments were performed using mouse protocols approved by the Animal Care and Use Committee of Johns Hopkins University School of Medicine (Baltimore, USA) and the Animal Care Committee of The Centre for Phenogenomics (Toronto, Canada). The mouse protocols used for this study are in accordance with the guidelines used by the National Institutes of Health (NIH) for mouse care and handling and the Canadian Council on Animal Care (CCAC).

**Table 1:** Sample sizes

Genotype	WT	*Kmt2d*^*+/βGeo*^	WT	*Kmt2d*^*+/βGeo*^
Treatment	Vehicle	Vehicle	AR-42	AR-42
Total sample size (n)	13	10	12	11
MRI (n)	11	9	10	10
Methylation microarray (n)	13	10	8	9
MRI and methylation microarray (n)	11	9	6	9

### DNA extraction

DNA was extracted from mouse blood stored at − 80 °C, using phenol–chloroform method. This was then repeated to purify DNA. PCR was run on genomic DNA using primers B-GeoF-(CAAATGGCGAT-TACCGTTGA) and B-GeoR-(TGCCCAGTCATAGCCGAATA), which are specific for the targeted allele to confirm genotype. Genomic DNA was then bisulfite converted using the EpiTect Bisulfite Kit (EpiTect PLUSBisulfite Kit, QIAGEN).

### DNA methylation microarray and statistical analysis

DNAm was assayed using the Illumina Infinium Mouse Methylation BeadChip (> 285,000 CpG sites) at The Center for Applied Genomics (TCAG), Hospital for Sick Children Research Institute, Toronto, Ontario, Canada in accordance with the manufacturer’s protocols. Samples were randomized on to array chips such that chips were balanced for genotype and treatment.

Mouse array data were background corrected and normalized in Genome Studio using Illumina normalization and then imported into R for further data preprocessing and analysis. CpG sites with a detection *p*-value greater than 0.05 were replaced with N/A, as well as sites covered by less than three beads. Probes mapping to mitochondrial DNA were also removed, but sex chromosomes probes were left in as all mice were male. A total of 284,394 CpGs remained following data cleaning.

Three outliers were identified by the detectOutlier function in the lumi package and were removed leaving a total of 40 samples for analysis: *n* = 13 vehicle-treated wildtype (vehicle WT), *n* = 8 AR-42-treated wildtype (AR-42 WT), *n* = 10 vehicle-treated *Kmt2d*^+*/βGeo*^ (vehicle *Kmt2d*^+*/βGeo*^), *n* = 9 AR-42-treated *Kmt2d*^+*/βGeo*^ (AR-42 *Kmt2d*^+*/βGeo*^) [20].

CpG sites differentially methylated by genotype, treatment, or phenotypic measures were identified using Limma regression, a modified linear regression run on log transformed beta values, M values [21, 22]. As blood is composed of multiple cell types with unique identities and therefore methylation patterns, 2 surrogate variables were included as covariates to account for major sources of underlying inter-individual variation [23, 24]. The number of significant SVs was determined by the “num.sv” function, using the Leek method and a model that included genotype or treatment, depending on which samples would be included in the model, and day of sacrifice [25]. SVs were generated using only the samples used in any given analysis. SVs were not included as covariates in the model in which total brain volume predicted DNAm. Day of sacrifice, i.e., 1 or 2 days following treatment, was also included as a covariate. Litter was excluded as there were no observable trends in DNAm between litters, as estimated by principal component 1 and 2 (PCs generated on all CpGs used in analysis; Additional file 1: Figure S1). CpGs reported as significant met genome-wide significance following False Discovery Rate (FDR) correction (*q*-value < 0.05) and a minimum effect size of mean group methylation difference above 5% (delta beta > 5%). For certain analyses, the *q*-value was relaxed to less than 0.1 as stated in text.

Differentially methylated regions (DMRs) were identified using combp function from the package ENmix [26]. Raw *p*-values from limma and CpG coordinates were fed into combp to identify regions at which *p*-values were measurably lower than surrounding sites. The FDR significance threshold for DMR identification was set to < 0.005. After DMRs were identified, they were manually subset to those containing at least 3 CpGs all with hypo- or hypermethylation, or at least 3 CpGs with hypo- or hypermethylation (if DMR contained more three CpGs and had bidirectional methylation changes). As well, each DMR had to contain at least one CpG found significant in the limma analysis.

### Brain imaging

Fixed brain samples were imaged using MRI to calculate regional volume [27]. A multi-channel 7.0 Tesla MRI scanner (Agilent Inc., Palo Alto, CA) was used to image the brains within their skulls. Sixteen custom-built solenoid coils were used to image the brains in parallel [28]. In order to detect volumetric changes, we used the following parameters for the MRI scan: T2- weighted, 3-D fast spin-echo sequence, with a cylindrical acquisition of k-space, a TR of 350 ms, and TEs of 12 ms per echo for 6 echoes, field-of-view equaled to 20 × 20 × 25 mm^3^ and matrix size equaled to 504 × 504 × 630. Our parameters output an image with 0.040 mm isotropic voxels. The total imaging time was 14 h [29].

### MRI registration and analysis

To visualize and compare any changes in the mouse brains the images are linearly (6 followed by 12 parameter) and nonlinearly registered together. Registrations are performed with a combination of mni_autoreg tools and ANTS (advanced normalization tools) [30–32]. In this process, scans are resampled with the appropriate transform and averaged to create a consensus population atlas representing the average anatomy of all brains. The result of the registration is to have all images deformed into alignment with each other in an unbiased fashion. This allows for the analysis of the deformations needed to take each individual mouse's anatomy into this final atlas space, with the ultimate goal being to model how the deformation fields relate to genotype [33, 34]. For a voxelwise analysis, the jacobian determinants of the deformation fields are calculated as measures of volume at each voxel. Regional differences can be calculated by warping a pre-existing classified MRI atlas onto the population atlas, which allows for the volume of 282 different segmented structures encompassing cortical lobes, large white matter structures (i.e., corpus callosum), ventricles, cerebellum, brain stem, and olfactory bulbs to be assessed in all brains [35–37]. Multiple comparisons in this study were controlled for using FDR [38].

## Results

### ***Kmt2d***^+***/βGeo***^ mouse brain volume and body weight

**Fig. 1:**
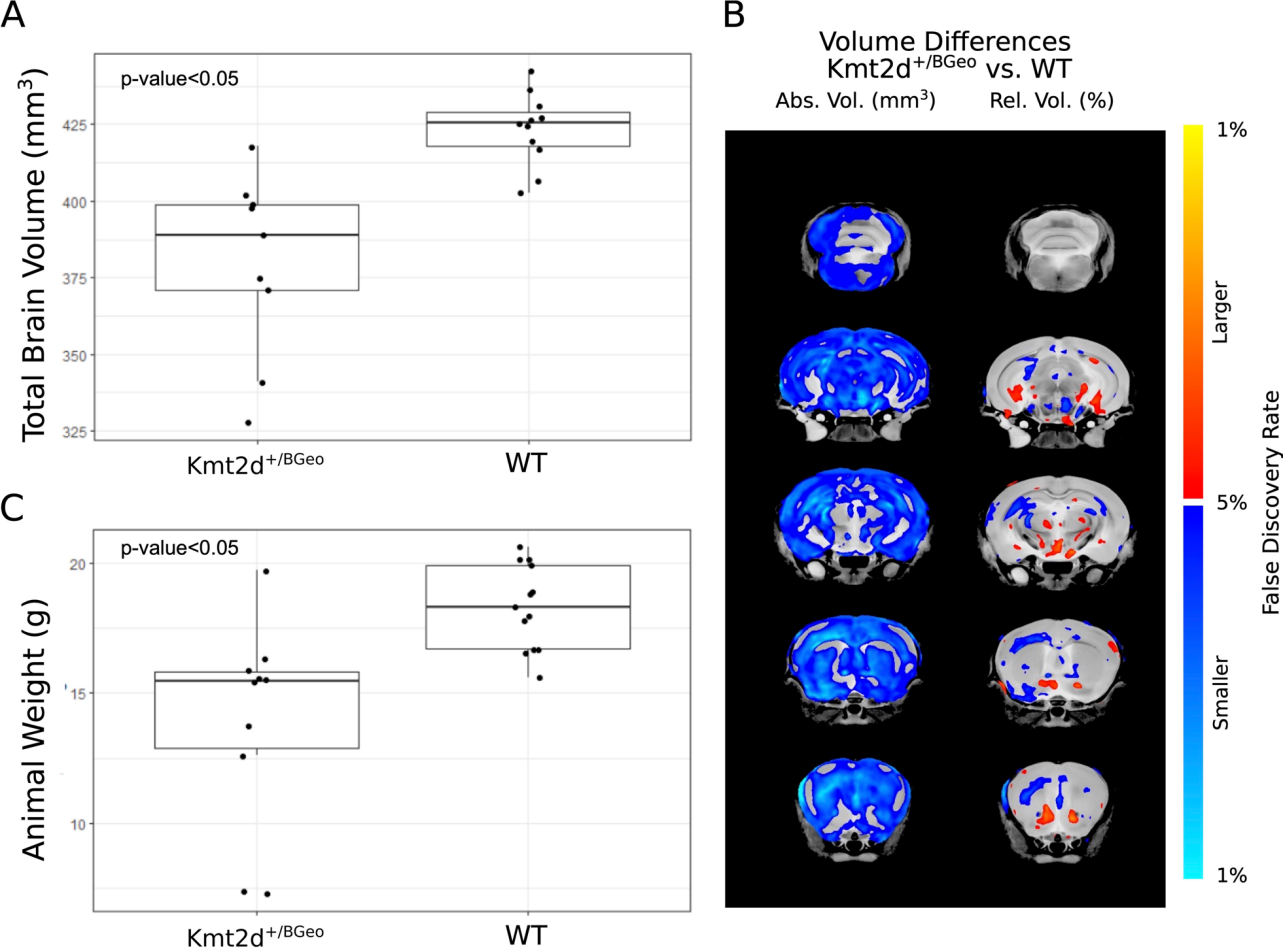
Size and brain volume deficits in Kmt2d^+/βGeo^ mice. **A** Body weights (g) of vehicle-treated animals at sacrifice. Kmt2d^+*/βGeo*^ animals were significantly smaller than WT (*t*-test *p*-value < 0.05).** B** Absolute (left) and relative (right) volume changes in Kmt2d^+*/βGeo*^ for all structures with *q*-value < 0.05. As observed in left column, volume loss is consistent across brain structures. **C** Total brain volume (mm^3^) vehicle-treated animals. Kmt2d^+*/βGeo*^ volumes were significantly smaller than WT (*t*-test *p*-value < 0.05)

*Kmt2d*^+*/βGeo*^ mice, carry an expression cassette encoding a β-galactosidase neomycin resistance fusion protein (β-Geo) in intron 50 of *Kmt2d *[4]. This allele produces a truncated protein that is lacking the SET domain, which confers methyltransferase activity to this protein. Forty-seven mice were born as 9 litters and assorted in sex and genotype matched pairs to minimize cage effects. Mice were weighed prior to sacrifice, at 6 weeks, and *Kmt2d*^+*/βGeo*^ mice (vehicle-treated; *n* = 10) were found to be significantly smaller than WT (vehicle-treated; *n* = 13; Fig. 1a). Growth retardation compared to WT littermates has previously been described in *Kmt2d*^+*/βGeo*^ mice at 5 months [4]. Of note, individuals with KS1 tend to have normal birth parameters but often exhibit failure to thrive and postnatal growth deficiency, in part due to feeding and gastrointestinal problems [3].

In additional to *Kmt2d*^+*/βGeo*^ mice having lower body weight, they exhibited significantly lower total brain volume at 6 weeks, as compared to WT (vehicle-treated groups; *n* = 9 *Kmt2d*^+*/βGeo*^ and *n* = 11 WT; *q*-value < 0.001; Fig. 1b). While total brain volume was decreased by 10% in the *Kmt2d*^+*/βGeo*^ group, specific regions with the largest differences include white matter (− 12.7%, *q* < 0.001), isocortex (− 12.4%, *q* < 0.001, and the olfactory areas (-11.3%, q < 0.01). As expected, brain volume was strongly correlated with body weight at sacrifice (Additional file 1: Figure S2). When relative volume was measured (regions as a percentage of total brain volume), the significance of some of these large areas was lost. However, more specific differences remained, including a relative volume decrease in the corpus callosum (− 5.4%, *q* = 0.01), and a relative increase in striatum (+ 7.25%, *q* = 0 < 0.001), the hypothalamus (+ 7.1%, *q* = 0.001), and the deep cerebellar nuclei (+ 3.3%, *q* = 0.04). There also was a trend found for a relative volume decrease in the overall isocortex (− 2.5% *q* = 0.07), indicating a larger cortical decrease comparatively to the whole brain volume. The hippocampus has previously been implicated in neurological phenotype of this mouse model and was found to be smaller in individuals with KS [4, 6, 39]; here, we found the hippocampal region and all underlying structures to have significantly smaller absolute volumes in *Kmt2d*^+*/βGeo*^ mice (*q* < 0.05). The hippocampal region and dentate gyrus showed volume differences of − 9.9% and − 11.4%, respectively. When relative volumes were taken into account hippocampal differences were localized to the stratum radiatum of CA1. Please see Additional file 2: Table S1 for a list of all structures investigated and corresponding *p*-values.

### Kabuki mouse DNA methylation signature overlaps with human KS1 signature and is enriched for genes with neurological functions

**Fig. 2:**
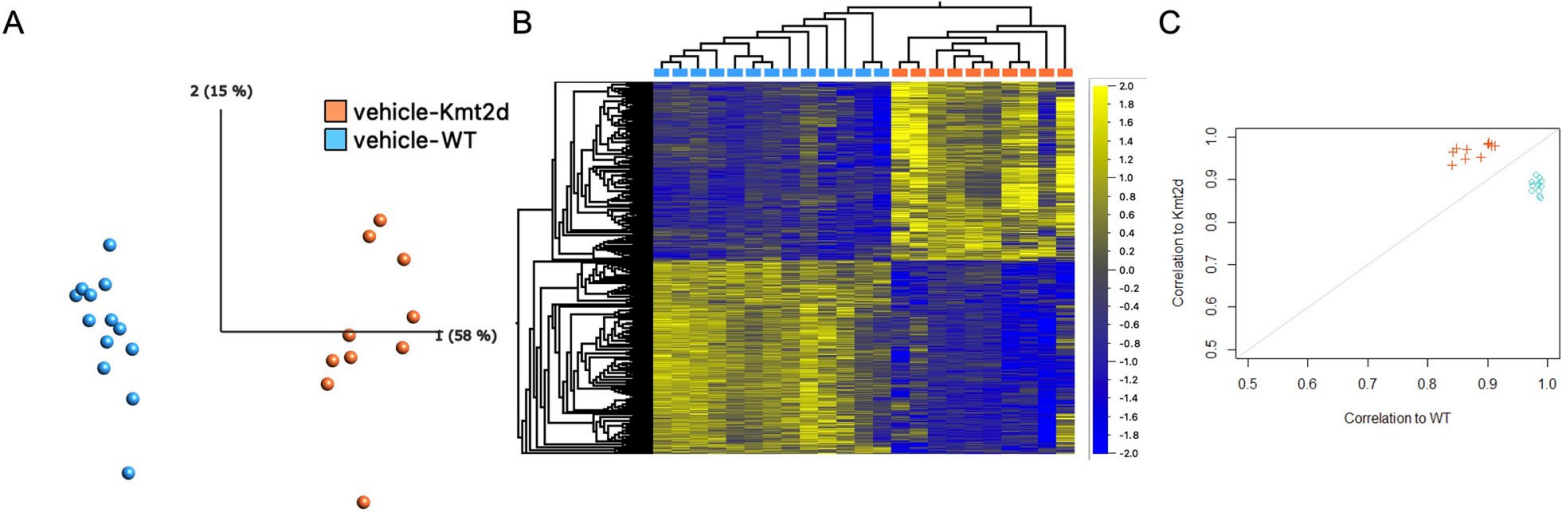
KS1 mouse signature has high sensitivity and specificity, as determined by cross-validation. **A** PCA at 1599 signature sites in only vehicle-treated samples (*n* = 13 WT; *n* = 10 KS1). **B** Heatmap of signature sites, showing even split between hyper- and hypomethylation, as well as clustering of samples by genotype. Dendrograms generated with Euclidean clustering. DNA methylation (Beta values) is normalized and ranges from − 2 to 2  **C** Leave-one-out cross-validation shows all samples correctly clustering by genotype. Samples, when left out and used for cross-validation, are more strongly correlated with samples of the genotype at signature sites

We compared the vehicle treated animals, *Kmt2d*^+*/βGeo*^ (*n* = 13) versus WT littermates (*n* = 10), to identify CpG sites differentially methylated by genotype. A linear model was applied to all CpGs sites on the array that passed quality control; 1599 CpGs met significance (*q*-value < 0.05; delta beta > 5%; Fig. 2a; Additional file 1: Figure S3; Additional file 2: Table S2), with 769 CpGs (48%) exhibiting hypermethylation in the *Kmt2d*^+*/βGeo*^ group, as compared to WT (Fig. 2b). This set of CpGs, hereafter referred to as a KS1 mouse “signature”, was tested for robustness using leave-one-out cross validation. The methylation patterns demonstrated 100% specificity and sensitivity, with all samples correctly predicted as WT or KS1 when validated on a signature generated on remaining samples (Fig. 2c).

Signature sites were also assessed for a difference in variances between genotypes as *Kmt2d*^+*/βGeo*^ mice may exhibit a general loss of control or dysregulation of DNAm patterns in addition to a directional shift in methylation. 73% of signature sites (1173) exhibited a larger range in the *Kmt2d*^+*/βGeo*^ group, as compared to WT; however, this difference was only found to be significant in one CpG, which mapped to the gene body of *Ndst1* (cg35789886_BC11; Levene’s test *q* < 0.05; Additional file 2: Table S2).

**Fig. 3:**
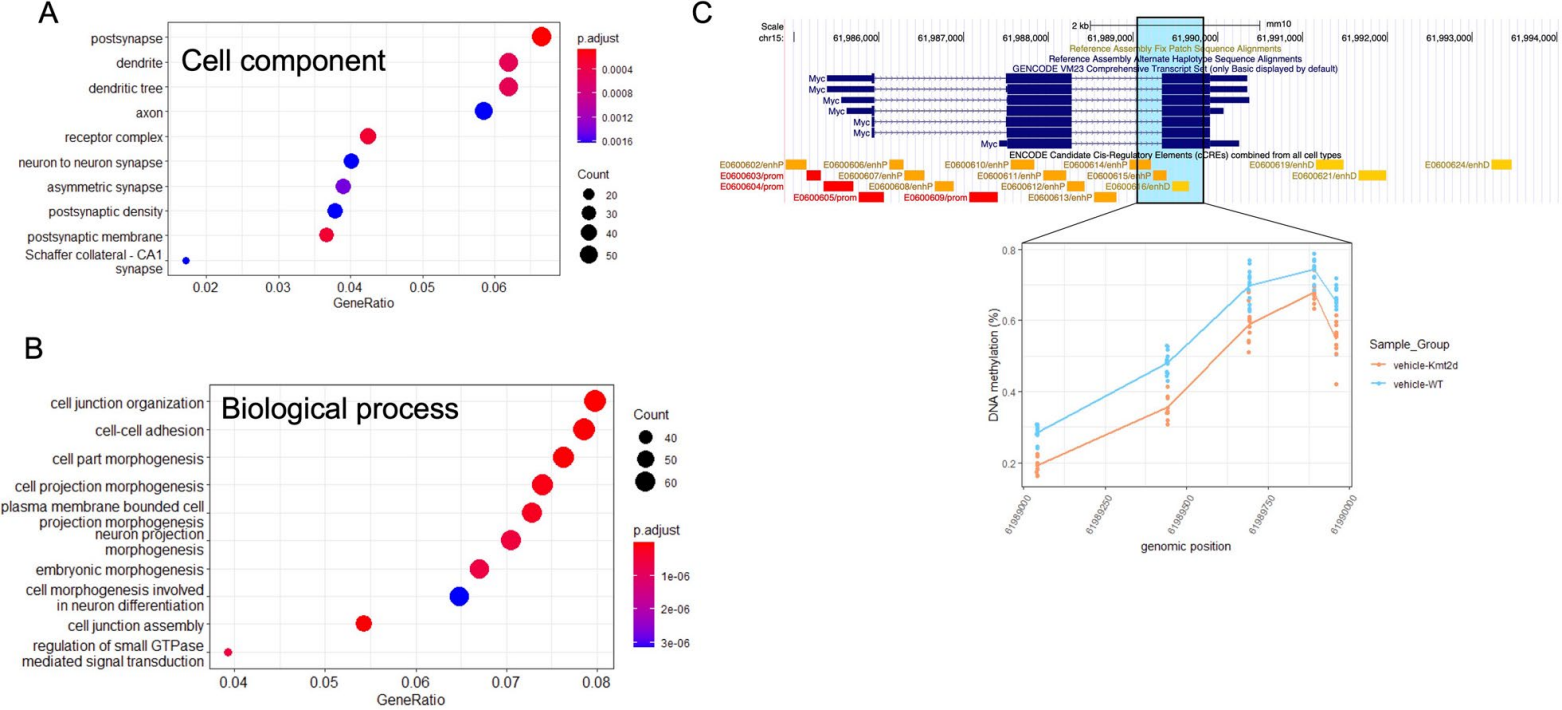
KS1 mouse signature sites mapped to genes related to KS1 phenotype. **A** GO enrichment top ten enriched cell component terms are specific to neuronal cells. **B** GO enrichment top ten enriched biological process terms are specific to neuronal cells. **C** DMR in the last exon of transcription factor *MYC*, composed of 5 CpGs, and overlaps proximal- and distal-acting enhancers

GO enrichment analysis was applied to signature sites, both hyper- and hypomethylated in KS1 mice, and significant cell component terms (*q*-value < 0.01) were specific to neuronal cells and included dendrite, post synapse and axon, while common biological process terms and molecular function terms included those related to cell morphogenesis and kinase activity, respectively (Fig. 3a and b; Additional file 1: Figure S4). We also compared signature sites to loci previously implicated in KS1, either in humans or in the KS1 mouse model presented here. Butcher et al. [12] published a blood KS1 signature on 11 individuals with KS1, which contained 221 CpG sites [12]. Nineteen genes were found to map to differential methylation in both the human and mouse signature (Table 2). Of those 19 genes, 15 genes exhibited methylation changes in the same direction in KS1 samples. A number of genes had described functions in neuronal tissue or were associated with autosomal dominant phenotypes in humans with neurodevelopmental or neurological features, including *SLITRK5, DLG4, ZMIZ1, RA1* and *VAC14*. Differential expression analysis was performed on *Kmt2d*^+*/βGeo*^ and WT littermates’ hippocampal cells and published in Zhang et al. [6]. There were 345 differentially expressed transcripts that met a *q*-value < 0.15, which mapped to 77 unique genes [6]. Five genes were found to be differentially expressed in *Kmt2d*^+*/βGeo*^ hippocampi and differentially methylated in *Kmt2d*^+*/βGeo*^ blood: *Ddx39b*, *Jarid2*, *Prph*, *Tyro3*, *Snhg1*.

**Table 2:** Overlapping differential methylation in mouse and human KS1 signatures

Mouse gene	Mouse signature sites	Mouse direction	Human gene	Human signature sites	Human direction	Agreement
*Ado*	1	Hypo	*ADO*	2	Hypo	Yes
*Dlg4*	1	Hyper	*DLG4*	1	Hypo	
*Hlx*	1	Hypo	*HLX*	1	Hypo	Yes
*Hnrnpa1*	2	Hypo	*HNRNPA1*	1	Hypo	Yes
*Il17re*	2	Hypo	*IL17RE*	1	Hyper	
*Kirrel3*	1	Hypo	*KIRREL3*	1	Hyper	
*Ksr1*	1	Hyper	*KSR1*	1	Hyper	Yes
*Rab11fip3*	1	Hypo	*RAB11FIP3*	1	Hypo	Yes
*Rai1*	1	Hyper	*RAI1*	1	Hyper	Yes
*Rps8*	3	Hypo	*RPS8*	1	Hypo	Yes
*Rrp12*	1	Hypo	*RRP12*	2	Hypo	Yes
*Sema6b*	3	Hypo	*SEMA6B*	1	Hypo	Yes
*Sh3rf3*	1	Hypo	*SH3RF3*	2	Hypo	Yes
*Slitrk5*	1	Hyper	*SLITRK5*	3	Hyper	Yes
*Slmap*	2	Hypo	*SLMAP*	1	Hypo	Yes
*Tnfaip2*	1	Hypo	*TNFAIP2*	3	Hypo	Yes
*Vac14*	1	Hyper	*VAC14*	1	Hypo	
*Zbtb46*	2	Hypo	*ZBTB46*	1	Hypo	Yes
*Zmiz1*	3	Hyper	*ZMIZ1*	4	Hyper	Yes

Finally, using the same set of vehicle-treated samples, we identified 99 differentially methylated regions (DMRs), each containing least three CpGs and at least one signature CpG (Additional file 1: Table S3). Similar to the direction of change observed in the signature, DMRs were evenly split between those containing hyper- vs. hypomethylated sites; specifically, 48 DMRs contained all hypermethylated CpGs; 49 DMRs contained all hypomethylated CpGs; and 2 DMRs contain CpGs with bidirectional changes. Multiple DMRs mapped to genes with functions that may relate to KS1 pathophysiology; these included the transcription factor *Myc* (Fig. 3c); protocadherin *Pcdh7*; GABA type B receptor *Gabbr2*; interferon regulatory factor *Irf6*; and synaptic vesicle glycoprotein *Sv2c*. As well, one DMR was intronic to *Jarid2,* which is differentially expressed in *Kmt2d*^+*/βGeo*^ hippocampi and one DMR mapped to *Slitrk2*, encoding a membrane protein expressed predominantly in neural tissue, which is differentially methylated in the blood of individuals with KS1.

### Kabuki mouse DNA methylation signature sites predict brain volume

**Fig. 4:**
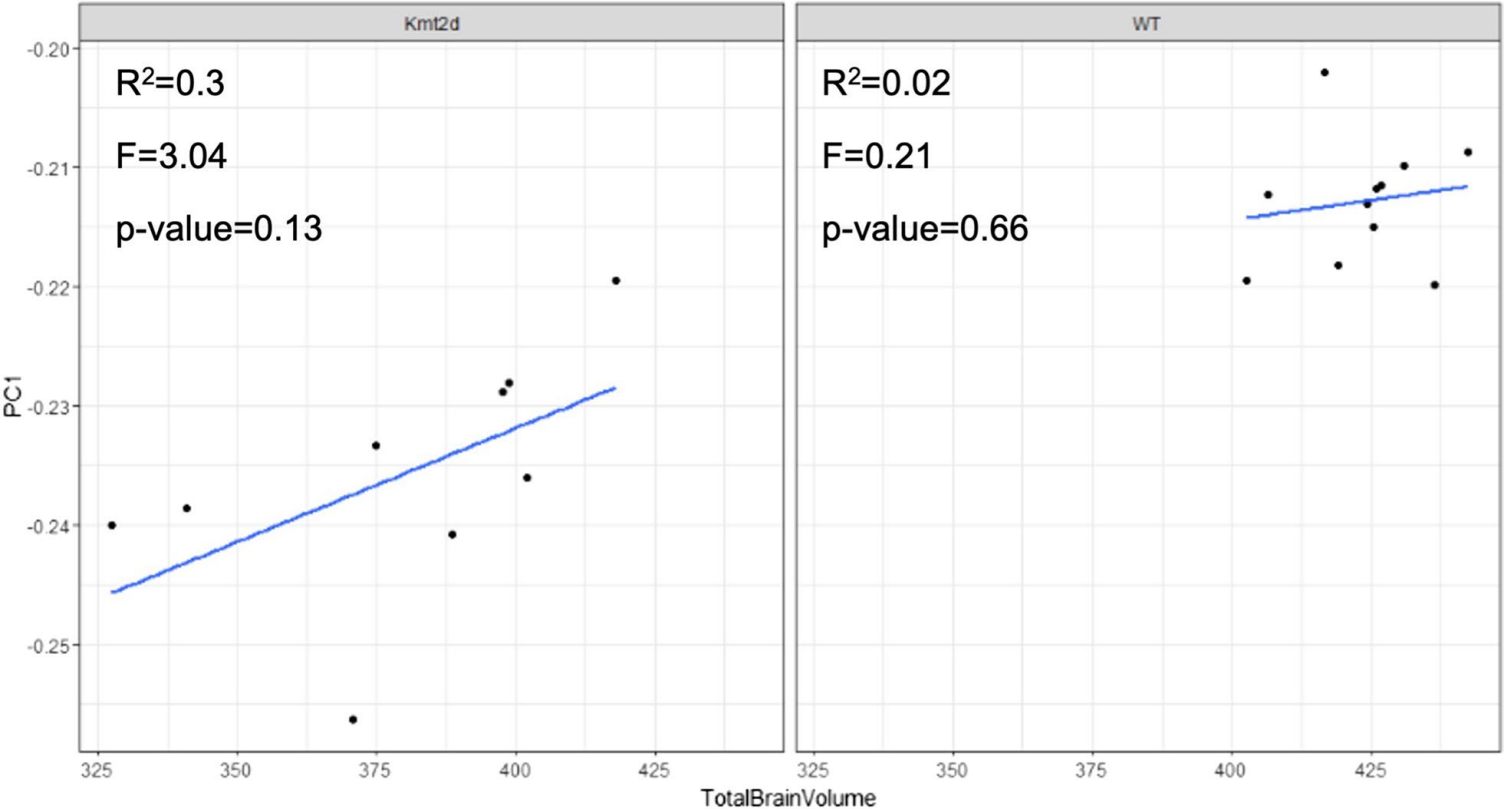
Linear relationship between brain volume and DNAm level at 27 CpGs in *Kmt2d*^+*/βGe*^ mice (left), as compared to WT mice (right). *Y*-axis represents PC1 scores of each animal, derived from PCA of 27 signature CpGs significantly associated with total brain volume. Blue lines represent line of best fit and statistics were generated from a linear regression performed within each group

We analyzed the vehicle-treated animals, *Kmt2d*^+*/βGeo*^ and WT, for a relationship between total brain volume and DNAm. Starting with the 1599 signature CpGs, we ran a model in which total brain volume was treated as the main effect and identified 27 significant CpGs (*q*-value < 0.05, delta beta > 5%; Additional file 1: Figure S5; Additional file 2: Table S4). A principal component analysis applied to these 27 CpGs revealed a clearer trend between DNAm and brain volume in the *Kmt2d*^+*/βGeo*^ mice, as compared to the WT, although this relationship did not meet statistical significance within either genotype (WT *p*-value < 0.66; *Kmt2d*^+*/βGeo*^ *p*-value = 0.13; Fig. 4). Nonetheless, the F-statistic in *Kmt2d*^+*/βGeo*^ mice was 3.04, as compared to 0.21 in WT mice, indicating a trend between these blood DNAm patterns and brain volume that may meet significance with a larger sample size. This difference in the strength of the association between *Kmt2d*^+*/βGeo*^ mice and WT, may be related to the differing ranges of brain volumes between genotypes, i.e., across WT animals, there is a range of ~ 40mm^3^ in total brain volume (min. 402.7; max. 442.5; median 425.4), and across *Kmt2d*^+*/βGeo*^ animals, there is a range of ~ 90mm^3^ in total brain volume (min. 327.5; max. 417.9; median 388.7). Alternatively, this relationship between DNAm and brain volume may be specific to or stronger in *Kmt2d*^+*/βGeo*^ mice and a consequence of epigenetic dysregulation. Supporting this scenario, the animals most severely affected are seen to have both the smallest brain volumes and greatest changes in methylation levels.

### Effects of AR-42 treatment on brain volume and blood DNA methylation

Next, we compared the vehicle- and AR-42-treated *Kmt2d*^+*/βGeo*^ animals to identify any changes to methylation patterns and/or brain volume. AR-42-treated *Kmt2d*^+*/βGeo*^ did not significantly differ from vehicle-treated *Kmt2d*^+*/βGeo*^ in brain volume (all structures *q* value > 0.05, data not shown). For WT mice only, the effect of treatment appeared to have a marginal trend toward a decrease in size (− 3.4%, *p* < 0.03, *q* < 0.16). However, this same trend was not seen in the *Kmt2d*^+*/βGeo*^ mice. We also specifically tested for interactions between genotype and treatment by linear modeling (Region–Genotype * Treatment) and the only effect found was the Genotype effect. There were no significant differences in volume (absolute or relative) for treatment nor for the interaction of genotype and treatment.

**Fig. 5:**
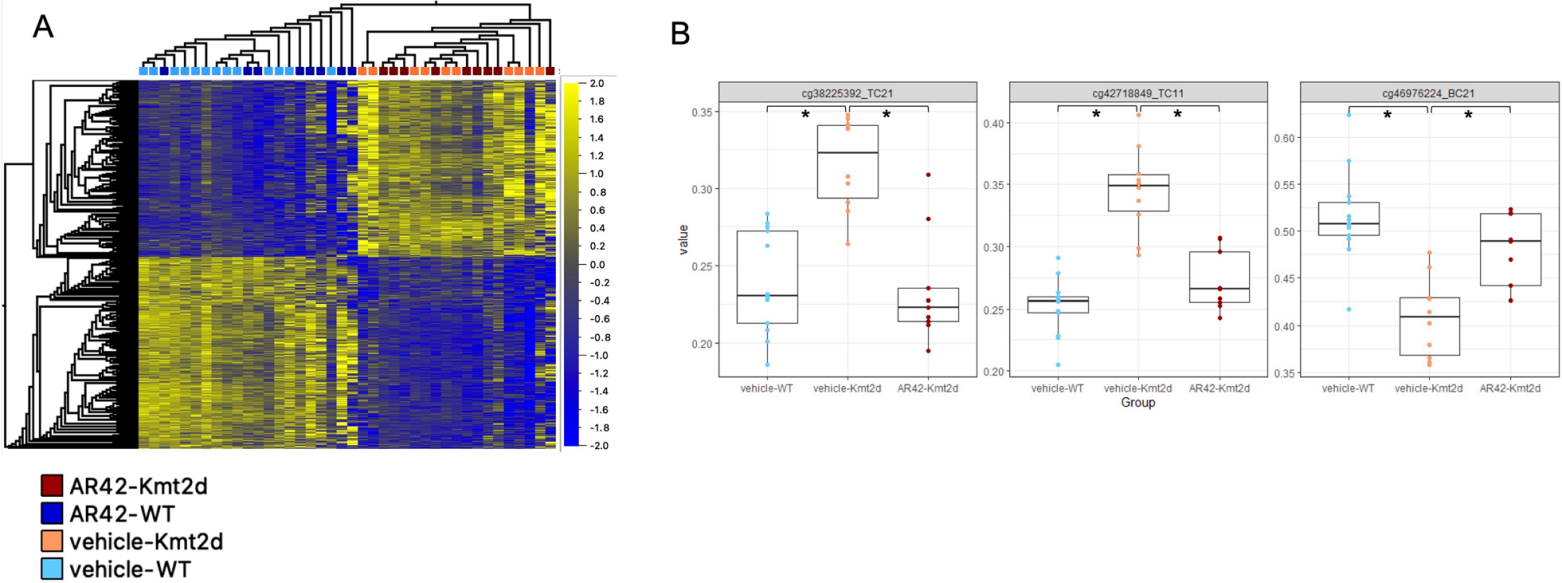
Ar-42 treatment *Kmt2d*^+*/βGeo*^ mice rescue a subset of signature CpGs. **A** Heatmap of signature sites with AR-42 treated samples included, showing that there is no broad methylation effect of treatment (as visualized by dendrogram, with treated samples intermixed with untreated but still clustering by genotype, providing further validation of the signature. **B** Three CpGs with significant methylation changes in AR-42 treated animals. Asterisks represent significant differential methylation (FDR *p-*value < 0.05) as tested by limma regression

Plotting the AR-42-treated animal’s DNAm values at the 1599 signature sites showed that treatment did not result in large, easily identifiable changes in methylation across the signature, as evidenced by the lack of clustering based on treatment (Fig. 5a). Nonetheless, this did confirm the efficacy of the signature in predicting genotype. To identify differential methylation between vehicle- and AR-42-treated *Kmt2d*^+*/βGeo*^ animals, we ran a linear model on the 1599 signature CpGs. Any significant sites would comprise those at which methylation levels were differentially methylated by genotype (i.e., dysregulated in vehicle-treated *Kmt2d*^+*/βGeo*^) and whose methylation status changed by AR-42 treatment (i.e., altered such that the treated KS1 mice had methylation levels more similar to untreated WT than untreated KS1 mice). This analysis of only KS1 mice, vehicle- vs. AR-42-treated identified 3 significant CpGs (Fig. 5b; Additional file 2: Table S5); these sites, at which methylation levels were partially rescued by AR-42-treatment, mapped to three genes *Fnbp1*, *Tpcn1*, *Hmgcll1*. While these genes have not been reported as differentially methylated in individuals with KS1, HMGCLL1, 3-hydroxymethyl-3-methylglutaryl-CoA lyase like 1, in mice and humans enables hydroxymethylglutaryl-CoA lyase activity which, as part of the ketone biosynthesis pathway, generates acetoacetate from hydroxymethylglutaryl-CoA. Acetoacetate is the precursor to 3-hydroxybutryate, an endogenous HDACi shown to have similar ameliorative effects on *Kmt2d*^+*/βGeo*^ phenotype as AR-42, when produced via a ketogenic diet [5].

Of the three “rescued” CpGs, two were originally hypermethylated in *Kmt2d*^+*/βGeo*^ and one was hypomethylated; this raised the question of whether AR-42 rescued only gain of methylation by promoting open chromatin with increased histone acetylation, leading to subsequent loss of methylation. We assessed this hypothesis by identifying all signature CpGs at which average AR-42-treated *Kmt2d*^+*/βGeo*^ DNAm was closer to average WT methylation than vehicle-treated *Kmt2d*^+*/βGeo*^. Unlike the previous analysis which identified 3 CpGs, we did not apply significance thresholds and assessed only mean group methylation. Of the 1599 signature CpGs, 1100 sites (69%) showed a correction of mean group methylation. This correction was agnostic to the original direction of the methylation change in vehicle-treated *Kmt2d*^+*/βGeo*^, as compared to vehicle-treated WT. In sites originally hypermethylated, 531/769 (69%) were rescued following AR-42 treatment. Similarly, in sites originally hypomethylated, 569/830 (69%) were rescued following AR-42 treatment. Therefore the majority of KS1 signature sites demonstrated a non-significant shift toward WT DNAm levels following 2 weeks of AR-42 treatment.

## Conclusion

We identify a blood DNAm signature in a KS1 mouse model, *Kmt2d*^+*/βGeo*^, which contains CpGs sites at which methylation levels correlate with phenotypic severity and are partially rescued by AR-42 treatment. This signature overlaps with the human KS1 signature at 19 genes, with the majority exhibiting shared directional changes between the two species [12]. The differentially methylated loci are also notable in that they mapped to genes that exhibited differential expression in the hippocampus of *Kmt2d*^+*/βGeo*^ mice [6]. Together these findings suggest that DNAm signatures carry valuable information related to pathophysiology and treatment but also that they may serve as biomarkers of treatment efficacy. AR-42 has been shown to have a positive effect on learning and memory deficits in *Kmt2d*^+*/βGeo*^ mice and also rescues loss of H3K4me3 in the hippocampus [4]. Here, we find a subset of signatures CpGs with significant changes in DNAm levels in AR-42-treated KS1 mice. Furthermore, we find that the majority of CpGs in the signature showed a change in methylation levels indicative of rescue, i.e., the *Kmt2d*^+*/βGeo*^ treated group has closer methylation levels to WT than untreated *Kmt2d*^+*/βGeo*^ mice.

One interesting feature of the mouse KS1 DNAm signature is the direction of methylation change in *Kmt2d*^+*/βGeo*^. One current hypothesis as to the etiology of KS1 is that the loss KMT2D activity results in an excess of closed chromatin. This is also thought to occur in Kabuki type 2, which is caused by pathogenic variants in KDM6A, a histone demethylase that removes the repressive histone marks H3K27me2/3. Their dysregulation results in highly overlapping clinical presentations, i.e., Kabuki type 1 and 2, and DNAm signatures [12, 13]. While previously assayed changes in histone marks in the *Kmt2d*^+*/βGeo*^ mouse support the closed chromatin theory, the DNAm changes do not directly match. One might expect a signature composed of mostly or entirely hypermethylated sites, denoting higher levels of closed chromatin and transcriptional repression; however, both human and mouse KS1-associated loci are composed of both hypo- and hypermethylated sites [12, 14]. This brings into question whether the KS1 DNAm pattern is a direct downstream effect of the loss H3K4 methylation or whether it also encompasses compensatory mechanisms resulting from epistatic mechanisms in the cell. It also highlights our dated view of DNAm as a repressive mark, which is often an oversimplification of a complex biological state, and assumptions that epigenetic cross-talk in disease states is not broken down.

While we observed common DNAm aberrations between the KS1 mouse model and individuals with KS1, an important distinction to take into consideration is the lack of the genetic variation across animals. The only genetic contribution to our derived mouse KS1 signature is the *Kmt2d*^+*/βGeo*^ variant. In humans, it is possible that both variant type in the *KMT2D* and genetic background impact both clinical presentation and DNAm levels at the KS1 signature. For example, missense variants causative of KS1 commonly occur in the SET domain and PHD-type zinc fingers #3 and #4 [40], while a number of reported missense variants in exons 38 and 39 of *KMT2D* cause a distinct multiple malformations disorder [41]. A small number of phenotype-genotype correlations have been reported in KS1 and further research is required to determine whether these differences translate into measurable differences in DNAm patterns for this disorder [42, 43]. Of note, despite the fact that both our WT and KS1 mice carry no background genetic variation, within each group we observed variability in DNAm patterning in the blood and phenotypic expression, as measured in animal weight and brain volume (Fig. 1; Fig. 4). With respect to disease pathophysiology, this finding supports the hypothesis that some phenotypic differences observed across humans with KS1 may not be related to variant type or genetic background but rather to regulatory changes via epigenetic modifications leading to downstream transcriptional differences.

While there are no significant effects of AR-42 on brain volume in *Kmt2d*^+*/βGeo*^ mice following a two-week treatment course, there are strong genotype effects on brain volume, i.e., *Kmt2d*^+*/βGeo*^ displays a total brain volume about 10% smaller than WT. A total brain volume change of this magnitude is common in autism-related mouse models, and being that these models are quite heterogeneous, they have a wide range in total brain volume differences [44]. Effect size differences in total brain volume in autism-related mouse models can range from -4 to + 6, and therefore an effect size difference seen here of -3.59 is quite large. These differences are likely connected to developmental delay and/or an arrest of growth. Moreover, microcephaly is considered a supportive clinical feature in the diagnosis of KS1. Relative volume differences give us a further assessment of localized differences comparatively across the brain. Overall, the limbic system seems to be strongly affected in the KS1 mouse model, as the anterior cingulate cortex, pre- or parasubiculum, and hypothalamus displayed significant changes in relative volume; although the hippocampus for the most part is unchanged in relative volume. The hippocampal differences are particularly interesting as previous reports have shown a loss in dentate gyrus volume compared to overall brain weight [4], and while we see an overall decrease of -11% in the dentate gyrus, when compared to overall brain volume that difference becomes insignificant. While that study also did not find a significant difference in overall brain weight, which is inconsistent with our brain volume decrease of 10%, a trend of lower brain weights was observed. The differing reports could be partially explained by the method with which they are measured (i.e., weight vs. volume and immunohistochemistry vs. MRI) and/or the ages at which measurements were taken, but need further study to clarify. Although similar studies in individuals with KS1 is limited, decreased volume of grey matter in KS1 has been reported; volume difference were localized to the bilateral precentral gyrus and middle frontal gyrus [39]. Neuroimaging on individuals with KS1 has also identified structural brain malformations including, abnormal pons and uplifted and hypoplastic vermis [45]. Taken together, these data suggest that while the hippocampus is still a structure of interest for elucidating neurological KS1 features, changes in other structures and both grey and white matter are likely consequential and merit further investigation.

We find brain volume to be significantly associated with 27 signatures sites. Interestingly, the DNAm changes at these CpGs display a linear relationship with brain volume in KS1 mice, but not WT mice, i.e., these associations are specific to the *Kmt2d*^+*/βGeo*^ genotype, and support their potential role as biomarkers of KS1 phenotypic severity. In humans there are several or emerging reports of DNAm levels within a signature corresponding to phenotypic severity. For example, Au-Kline syndrome is a neurodevelopmental disorder caused by pathogenic variants in *HNRNPK*; in 11 of 22 individuals with an Au-Kline diagnosis, tested on the DNAm signature were found to be “intermediate” meaning that their DNAm at signature sites fell between levels that matched individuals carrying *HNRNPK* loss-of-function variants and levels in healthy controls or individuals with benign variants [46]. Interestingly, these intermediate methylation patterns were primarily identified in individuals who (1) carried an *HNRNPK* missense variant and (2) presented with a milder clinical presentation, thus indicating a genotype-phenotype-epigenotype correlation [46]. Similarly, in the 22q11.2 deletion syndrome (22q11DS) DNAm signature, individuals carrying a deletion distal to the canonical 22q11.2 deletion, were found to be “control-like” in their DNAm levels and distinct from patterns in individuals with the larger, canonical 22q11.2 deletion [47]. While phenotypes were not available for these individuals, the distal 22q11.2 deletion has been previously associated with a milder phenotype [48]. While both of these examples highlight genotype-phenotype-epigenotype correlations, it is important to note that in the KS1 mice, we observe a phenotype-epigenotype correlation between brain volume and DNAm, independent of genotype as these mice are genetically identical. There are numerous monogenic neurodevelopmental disorders, including 22q11DS, Snijders Blok-Campeau syndrome (*CDH3*) and KBG syndrome (*ANKRD11*), with reports of discordant monozygotic twins and/or variable expressivity across individuals in families who inherit the same pathogenic sequence variant [49–51]. Moreover, a large-scale study of exome and SNP-array data from ~ 700,000 individuals identified rare loss-of-function and predicted damaging variants in genes associated with monogenic disorders in individuals with subclinical neurodevelopmental phenotypes; these findings highlight further the unmeasured and underreported variable expressivity in this group of disorders [52]. While genetic background and in utero environmental factors contribute to incomplete penetrance, epigenetic mechanisms are likely to have an effect and the possibility of identifying epigenetic patterns corresponding to this clinical variability would fill a large knowledge gap in our understanding of pathophysiology [53].

DNAm signatures have proven valuable in the diagnostic realm, although with any emerging field there are still many questions to address, such as the extent to which DNAm changes in the blood are representative of those in the brain or other tissues. It is also unclear how and when a DNAm signature is established during development. It is also important to consider if the tissue tested demonstrates its own phenotype, for example, individuals with KS1 commonly face immune dysfunction including reduced B cell population [42]. Furthermore, KMT2D has a role in regulating B cell development and its disruption facilitates lymphomagenesis [54]. Given this function in lymphocytes, it is plausible that the DNAm signature resulting for the *Kmt2d*^+*/βGeo*^ genotype is affected by these cell types. Kmt2d is expressed in the mouse embryo as early as the two-cell stage (as reported in the MGI database GXD), and so its dysregulation may disrupt DNAm long before cellular differentiation occurs [55, 56]. As such, we hypothesize that this signature likely contains differential methylation patterns (as compared to WT) that are specific to the cells measured, as well patterns that are shared across tissues. The latter may indicate downstream transcriptional dysregulation in other cell types, including neurons and glia. We plan to test this hypothesis with matched blood and brain tissue, which was not possible here as the brain was fixed for imaging.

In sum, this work illustrates how mouse model research into human neurodevelopmental disorders has great utility for understanding disease pathophysiology, and defining optimal treatment approaches as well as developing biomarkers of outcomes for this group of syndromes. While our future studies will undoubtedly benefit from “matched” -omics data, i.e., histone mark and transcriptional measures generated on the same animals in which we assess DNAm and brain volume, we have begun to uncover a clearer picture of KS1 pathophysiology by connecting these molecular marks. As well, given our positive findings for DNAm patterns serving as a biomarker of AR-42 treatment and potentially phenotypic severity, we plan to build on this study and measure DNAm and histone methylation in multiple tissues across the life course of KS1 mice.

### Supplementary Information

**Additional file 1**. Supplementary Figures S1–S5.

**Additional file 2**. Supplementary Tables S1–S5.

## Data Availability

Mouse brain volume data and DNA methylation data will be made available on GEO at the time of publication.

## Abbreviations

DNAm: DNA methylation
H3K4: Histone H3 lysine 4
HDACi: Histone deacetylase inhibitor
KS1: Kabuki syndrome type 1
MDEM: Mendelian disorder of epigenetic machinery
