# Supplementary material for: Peripheral blood DNA methylation and neuroanatomical responses to HDACi treatment that rescues neurological deficits in a Kabuki syndrome mouse model
Source: Clin Epigenetics. 2023 Oct 27;15:172. doi: 10.1186/s13148-023-01582-x (PMC10605417; doi:10.1186/s13148-023-01582-x)
Supplement: Supplementary file 1 — Additional file 1. Supplementary Figures S1–S5. [file 13148_2023_1582_MOESM1_ESM.ppt]

## Slide 1
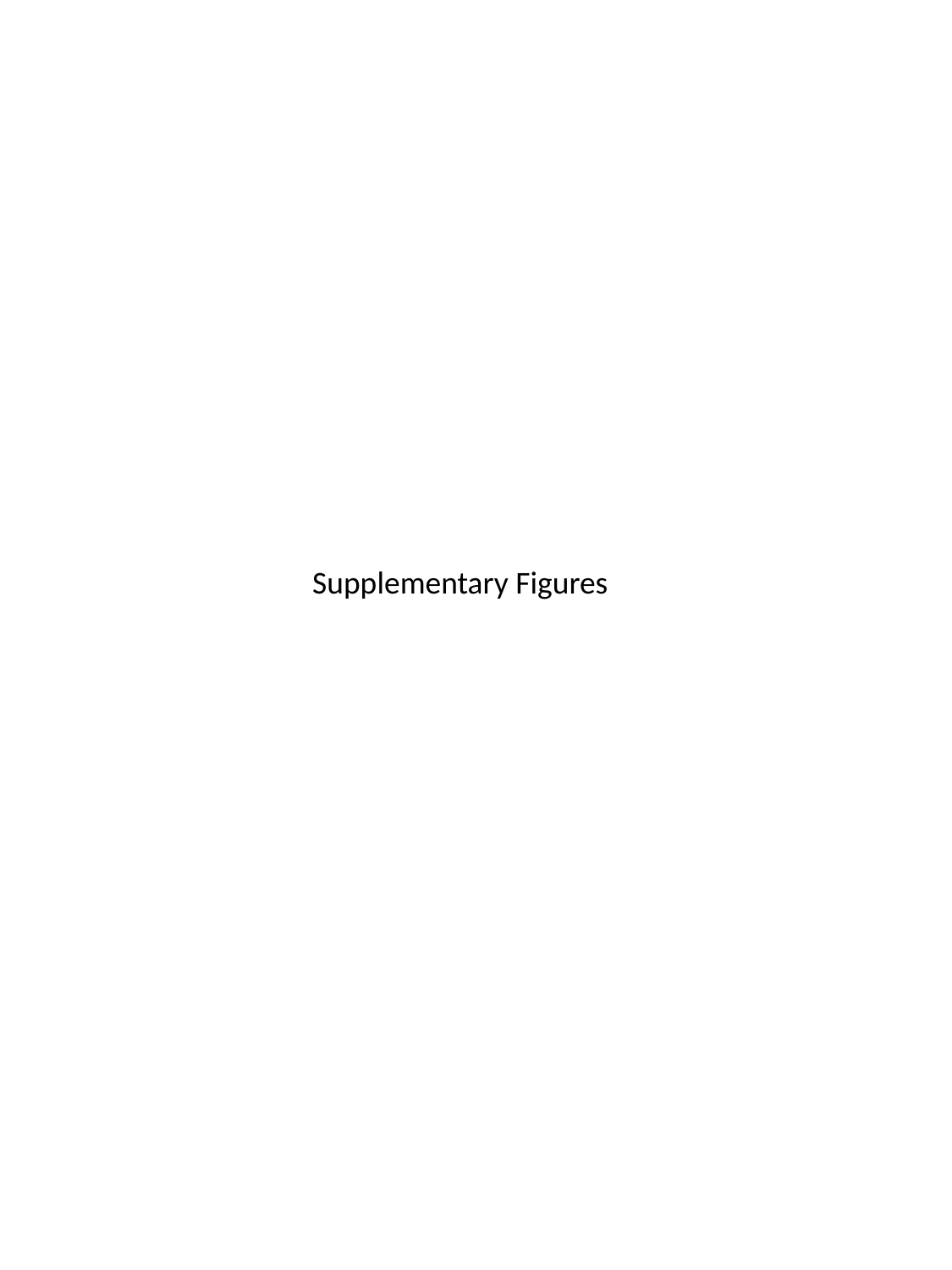

Supplementary Figures

## Slide 2
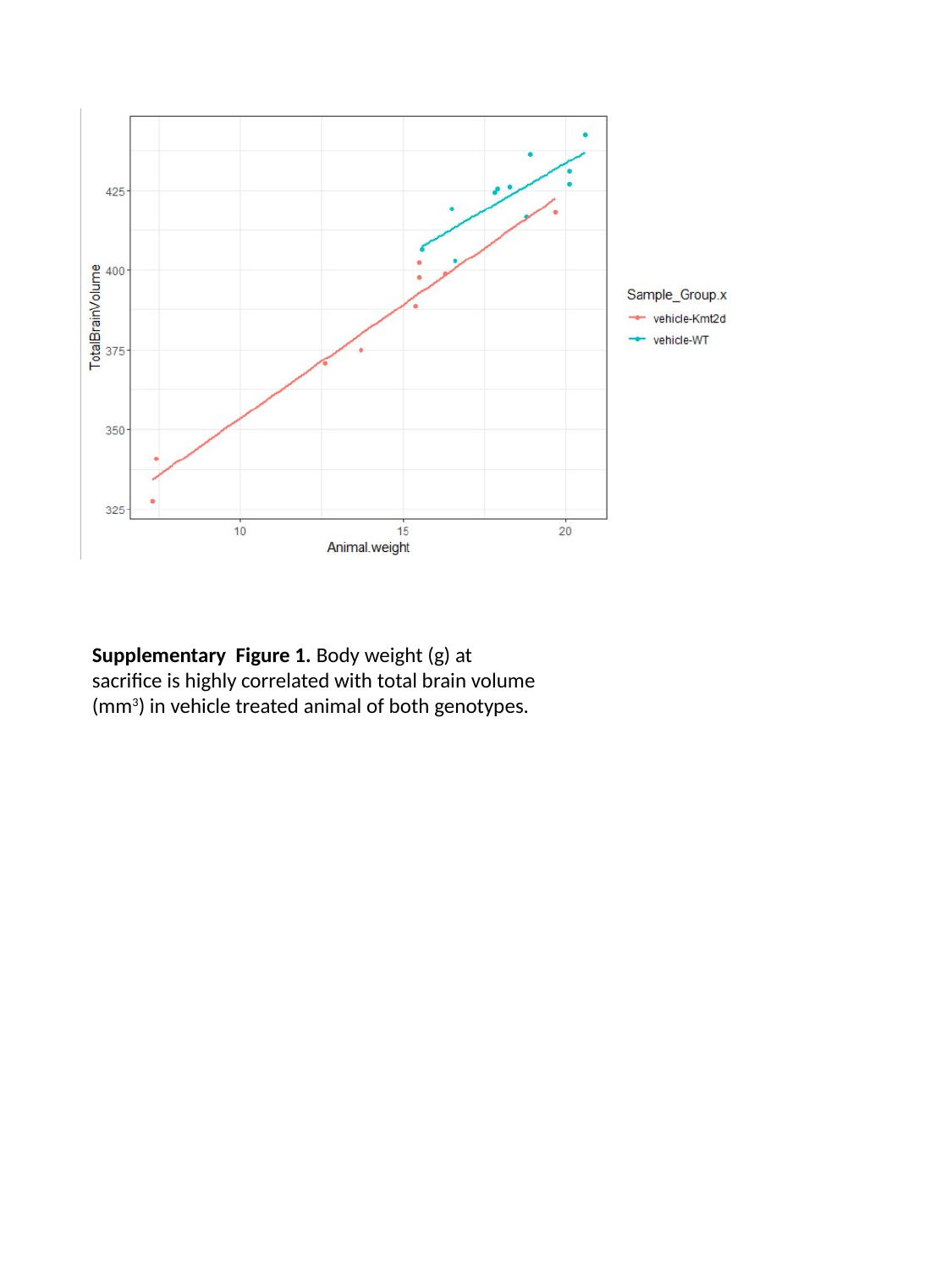

Supplementary Figure 1. Body weight (g) at sacrifice is highly correlated with total brain volume (mm3) in vehicle treated animal of both genotypes.

## Slide 3
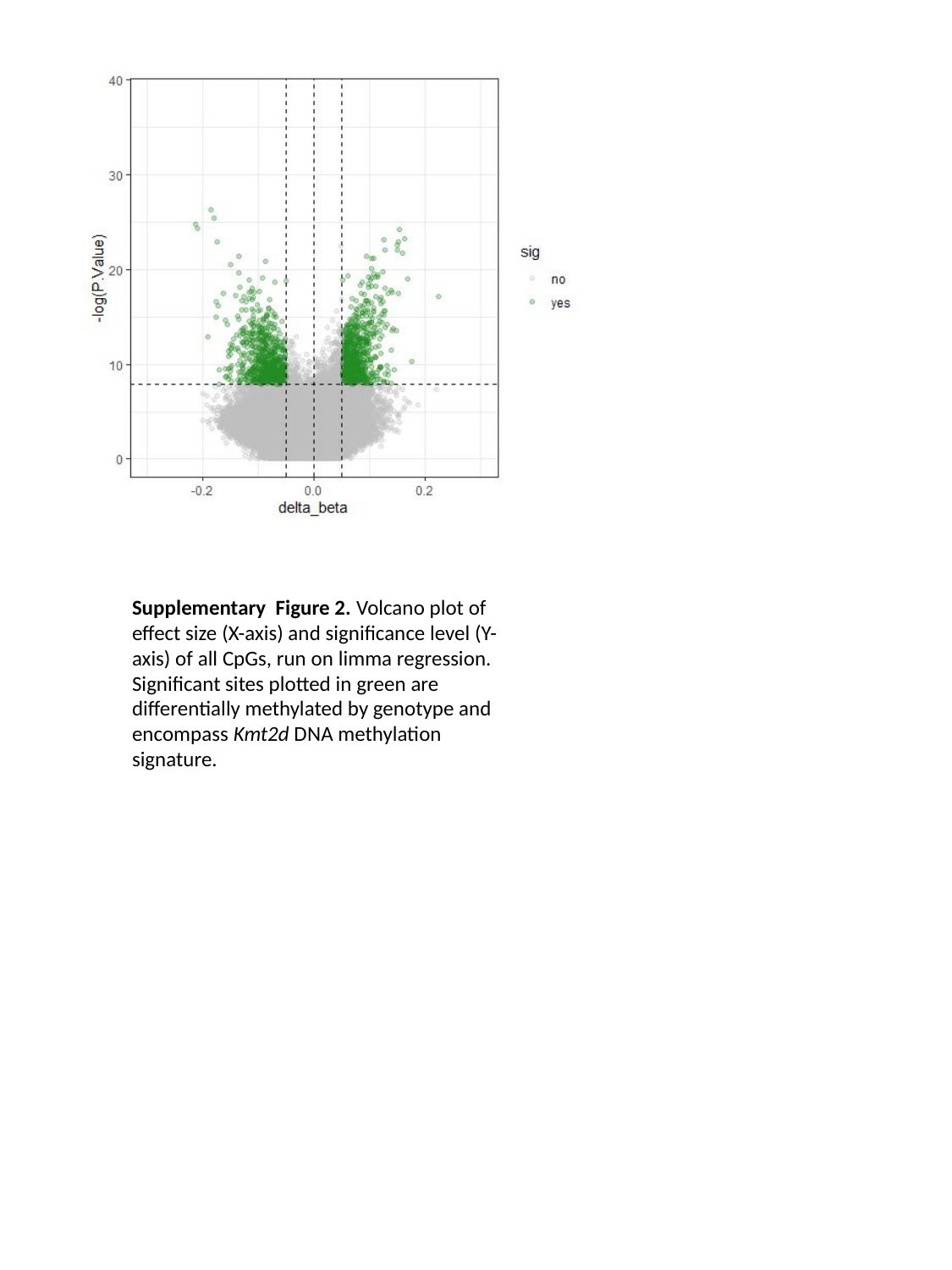

Supplementary Figure 2. Volcano plot of effect size (X-axis) and significance level (Y-axis) of all CpGs, run on limma regression. Significant sites plotted in green are differentially methylated by genotype and encompass Kmt2d DNA methylation signature.

## Slide 4
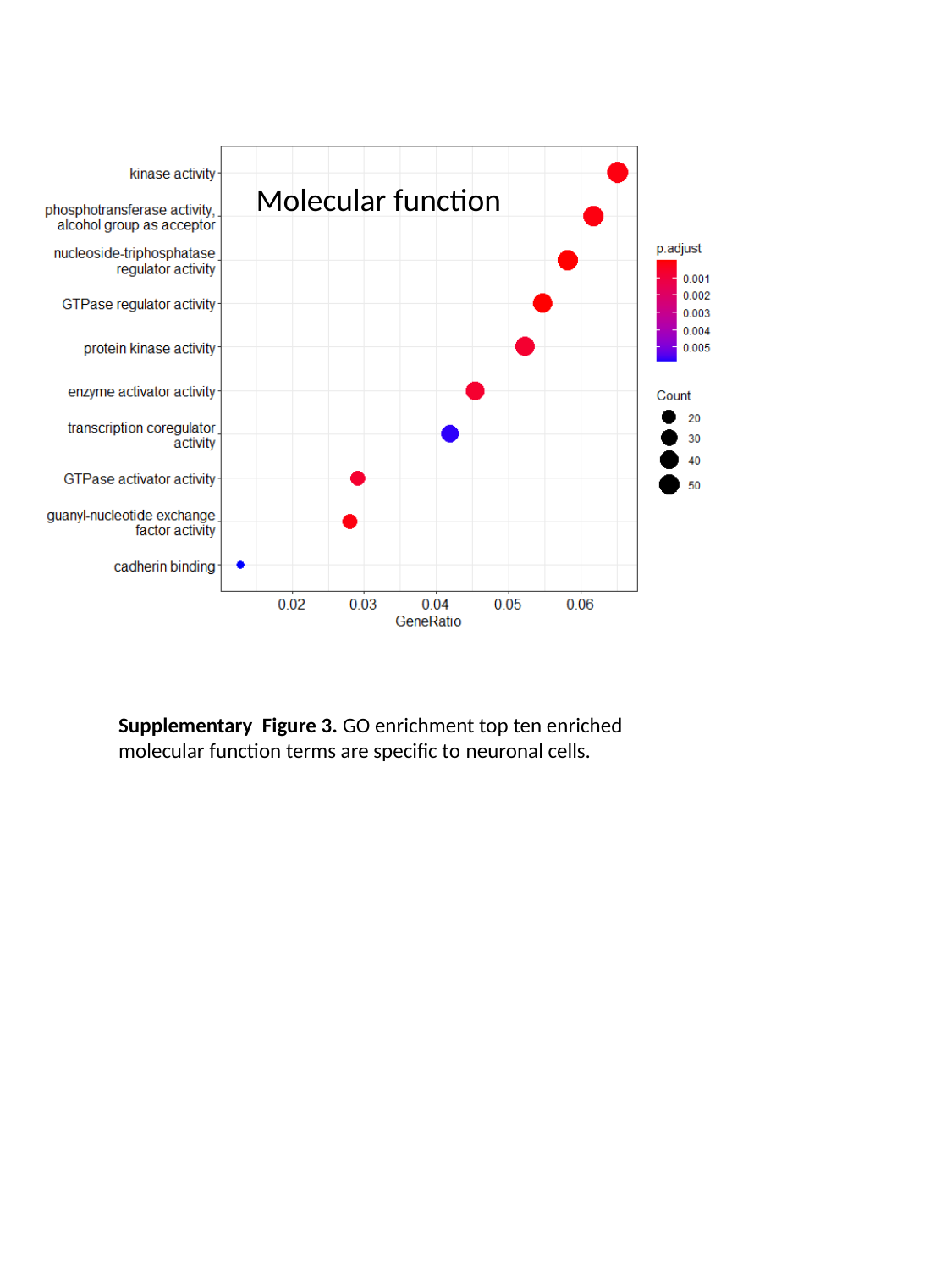

Molecular function
Supplementary Figure 3. GO enrichment top ten enriched molecular function terms are specific to neuronal cells.

## Slide 5
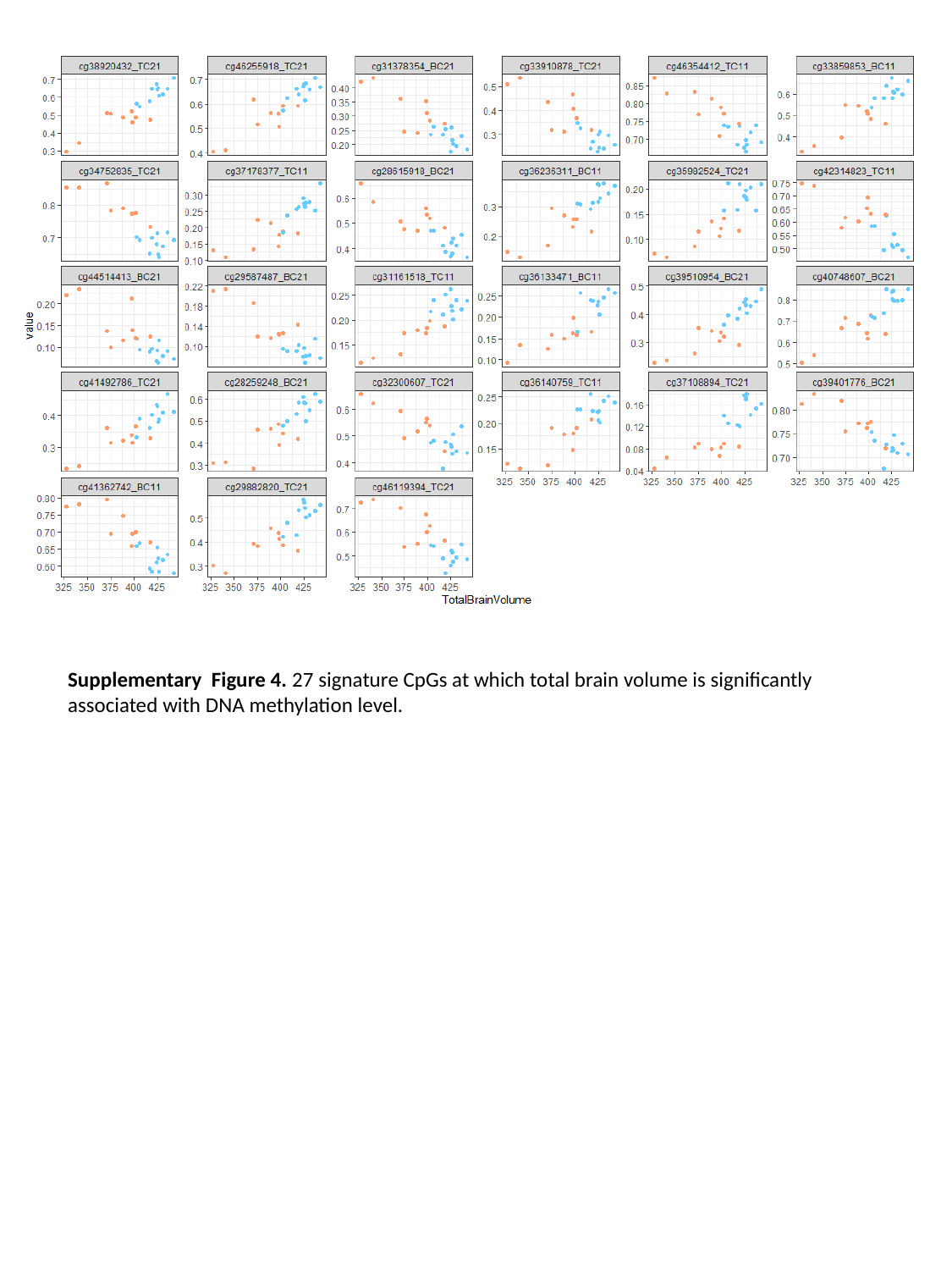

Supplementary Figure 4. 27 signature CpGs at which total brain volume is significantly associated with DNA methylation level.

## Slide 6
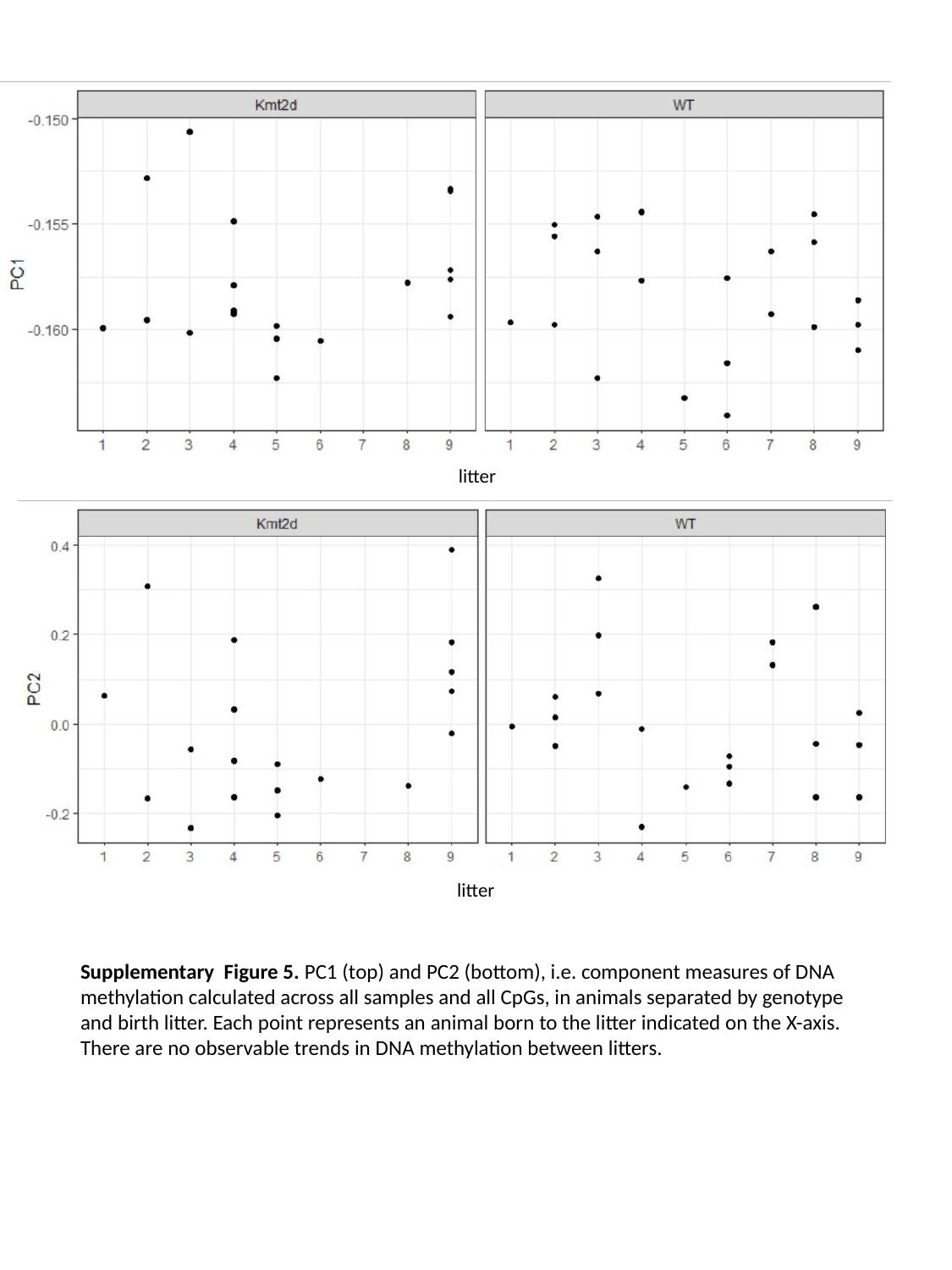

litter
litter
Supplementary Figure 5. PC1 (top) and PC2 (bottom), i.e. component measures of DNA methylation calculated across all samples and all CpGs, in animals separated by genotype and birth litter. Each point represents an animal born to the litter indicated on the X-axis. There are no observable trends in DNA methylation between litters.
